# Cocirculation of Human Monkeypox Virus Clade 1b with Varicella Zoster Virus, Uganda

**DOI:** 10.3201/eid3210.251264

**Published:** 2026-10

**Authors:** Nicholas Bbosa, Alfred Ssekagiri, Hamidah S. Namagembe, Stella E. Nabirye, Ronald Kiiza, Danstan Kabuuka, Stephen Balinandi, Tom Lutalo, John Kayiwa, Henry Kyobe Bosa, Robert Downing, Michael G. Berg, Mary A. Rodgers, Gavin A. Cloherty, Pontiano Kaleebu, Deogratius Ssemwanga

**Affiliations:** Uganda Virus Research Institute, Entebbe, Uganda (N. Bbosa, A. Ssekagiri, S.E. Nabirye, D. Kabuuka, S. Balinandi, T. Lutalo, J. Kayiwa, R. Downing, P. Kaleebu, D. Ssemwanga); Medical Research Council/Uganda Virus Research Institute and London School of Hygiene and Tropical Medicine Uganda Research Unit, Entebbe (N. Bbosa, H.S. Namagembe, R. Kiiza, P. Kaleebu, D. Ssemwanga); Abbott Pandemic Defense Coalition, Abbott Park, Illinois, USA (N. Bbosa, M.G. Berg, M.A. Rodgers, G.A. Cloherty); Uganda Ministry of Health, Kampala, Uganda (H.K. Bosa); Uganda People’s Defence Forces, Kampala (H.K. Bosa); Makerere University Lung Institute, Kampala (H.K. Bosa); Abbott Diagnostics, Abbott Park (M.G. Berg, M.A. Rodgers, G.A. Cloherty)

**Keywords:** viruses, sexually transmitted infections, human, mpox, monkeypox virus, varicella zoster virus, Uganda, cocirculation, co-infection, genotyping

## Abstract

In July 2024, we reported information on the initial human mpox cases in Uganda. Genomic characterization of clinically suspected cases (July–December 2024) revealed cocirculation of several monkeypox virus clade 1b clusters and varicella zoster virus. Our findings highlight the need to refine differential diagnoses by molecular techniques and strengthen surveillance.

In July 2024, human mpox cases were initially reported in Uganda (*1*). As of May 28, 2025, up to 6,479 confirmed mpox cases, including 44 deaths, have been reported (*2*). However, several clinically suspected mpox case-patients tested negative for monkeypox virus (MPXV) by real-time PCR. Mpox can be misdiagnosed as other infections that manifest in rash, especially during the early macule and papule stages of the infection (*3*). Patients with mpox and chickenpox co-infections have been reported in the Democratic Republic of Congo (DRC) (*4*), Burundi (*5*), and Nigeria (*6*). Mpox case-patients frequently experience a febrile prodrome with a high fever 1–4 days before the onset of rash, whereas a low-grade fever is more common in chickenpox patients (*4*). Furthermore, lymphadenopathy is a distinguishing symptom characteristic of mpox infection (*6*) but not chickenpox infection. Here, we describe phylogenetic characterization and mutational analysis of circulating MPXV and other viruses from clinically suspected mpox cases in Uganda.

We conducted real-time PCR on 1,072 samples from patients with clinically suspected MPXV during July–December 2024. A total of 193 persons tested positive for MPXV. Confirmed mpox case-patients were 48.7% male, 51.3% female; cases primarily occurred in persons 16–30 years of age (47.8%) (Appendix 1 Table 1). We obtained 65% of samples from central Uganda. Of the 879 (82%) samples that tested negative for MPXV by PCR, we sequenced a randomly selected subset (127 [14%]) to identify other potential pathogens.

We genotyped 115 MPXV PCR–positive samples using target enrichment NGS (Illumina, https://www.illumina.com; Twist Biosciences, https://www.twistbioscience.com) and the 127 MPXV PCR–negative samples using metagenomic NGS and target enrichment NGS, followed by sequencing on a MiSeq (*1*). We analyzed FASTQ files using UVRI’s in-house metagenomic NGS (*1*) and Abbott’s DiVir pipelines (*7*) and One Codex software (S.S. Minot et al., unpub. data, https://www.biorxiv.org/content/10.1101/027607v2). We performed phylogenetic tree reconstructions using IQ-TREE (*8*), which showed new Uganda MPXV sequences were clade 1b and genetically close to previous sequences from Uganda and DRC (Figure 1, panel A).

**Figure 1:**
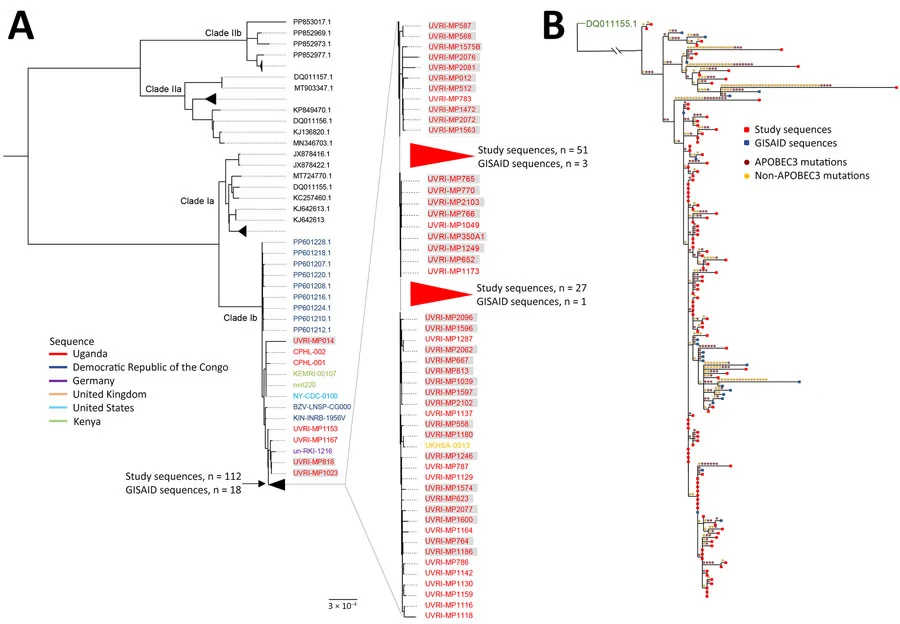
Phylogenetic and mutational characterization of Uganda monkeypox virus (MPXV) strains in study of cocirculation of human MPXV clade 1b with varicella-zoster virus, Uganda. A) Maximum-likelihood phylogenetic tree of MPXV sequences generated with 1,000 bootstrap resampling. Tree includes 115 new sequences from Uganda (highlighted with a gray rectangular background) and 37 clade 1B reference sequences retrieved from GenBank and GISAID (https://www.gisaid.org) (accession numbers provided). Highly similar sequences have been collapsed (denoted by triangle). Scale bar indicates number of nucleotide substitutions per site. B) New (n = 115; red squares) and previously reported (n = 22; blue squares) Uganda sequences were aligned, and Squirrel software was used to infer the maximum-likelihood phylogeny and map APOBEC (brown circle) and non-APOBEC (yellow circle) mutation events. The number of unique mutations from this study and shared mutations distinct from the DQ011155.1 clade I archetype are listed.

We performed a mutational analysis on Uganda strains (n = 137) using Squirrel software (https://artic.network/software/squirrel), comparing new sequences (n = 115) to previous sequences (n = 22) relative to reference strain Zaire-1979-DQ011155.1 from GenBank. Squirrel infers a maximum-likelihood phylogeny with IQ-TREE2 and performs ancestral state reconstruction to map APOBEC and non-APOBEC mutation events onto individual branches (Figure 1, panel B). TC->TT or GA->AA dinucleotide substitutions were consistent with APOBEC3-mediated cytosine deamination. The reconstructed phylogeny identified 284 mutation events (synonymous, nonsynonymous, intergenic) in Uganda sequences; of those, 118 (41.4%) mutations were APOBEC3-mediated (Appendix 1 Table 2). We observed distinct profiles of affected genes among sequences from this study compared with previously reported Uganda sequences (Appendix 2). Focusing on nonsynonymous changes, functional annotations of affected genes acquiring APOBEC and non-APOBEC mutations were similar between cohorts. For previous strains, fewer functional annotations were identified related to apoptosis inhibition, viral maturation, and mRNA (de)capping. For strains sequenced in this study, amino acid changes affected proteins involved in inhibition of host responses (inflammasome, T-cell activation) and immune evasion, transcription and RNA metabolism (poly-A polymerase, DNA-dependent RNA polymerase), virion morphogenesis, and viral entry/egress pathways.

We detected varicella zoster virus (VZV) in 106 (83%) of 127 specimens with genome coverage >80% (Figure 2, panel A). VZV sequences were genetically similar to those identified in Nigeria, Ghana, and Guinea-Bissau (Figure 2, panel B). Other pathogens identified included coxsackievirus A6 (9 [7%]), measles virus genotype B3 (7 [6%]), polyomaviruses (5 [4%]), *Cutibacterium acnes*, *Staphylococcus aureus*, *Streptococcus pyogenes,* and *Corynebacterium diphtheriae.* We submitted MPXV sequences (n = 115) to GISAID (https://www.gisaid.org, under EpiPox) in nonsequential installments (Appendix 1) and VZV data (National Center for Biotechnology Information BioProject no. PRJNA1281018) to GenBank (accession nos. PV845252–301).

**Figure 2:**
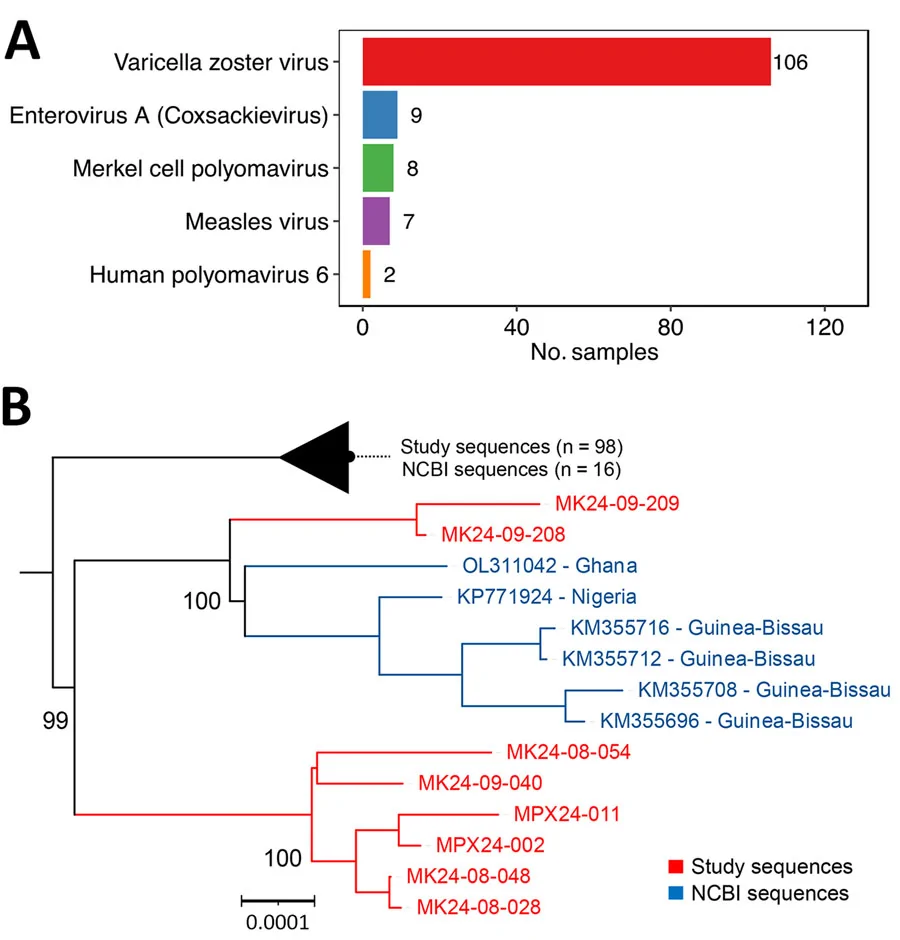
Distribution of viral pathogens and phylogenetic analysis in study of cocirculation of human monkeypox virus clade 1b with varicella-zoster virus, Uganda. A) Viral pathogens detected in monkeypox virus–negative samples by metagenomic next-generation sequencing. Bar plot shows the number of samples in which viruses were identified with a genome coverage >80%. B) Maximum-likelihood phylogenetic tree of varicella-zoster virus sequences. The tree includes 106 high-coverage genome sequences (>80% genome coverage) generated in this study and other publicly available complete genomes from NCBI (accession numbers provided). Most sequences have been collapsed into the triangle at the top, which contains 98 varicella zoster virus sequences generated in this study and 16 publicly available Uganda genomes, together forming a single well-supported clade. Sequences shown individually are those resolving outside that clade: 8 study sequences from Uganda (red), which form 2 distinct lineages, and the 6 publicly available genomes from Ghana, Nigeria and Guinea-Bissau (blue) that are their closest relatives. NCBI, National Center for Biotechnology Information.

Genotypic characterization of confirmed mpox cases determined that MPXV sequences belonged to clade Ib and were highly related to previously reported Uganda sequences (*1*), although mutational profiles displayed minimal overlap. Gene ontology suggests recent strains are under selective pressure to evade host responses and increase transmission through improved replicative and transcriptional fitness. Evidence from Kenya (*9*) and Ethiopia (*10*) indicates that varicella is frequently misdiagnosed as mpox, consistent with DRC (*4*,*11*) and Burundi (*5*) studies that identified VZV in 20 (67%) of 30 samples from clinically suspected persons who tested PCR-negative during an mpox outbreak (*5*). Our study reinforces that mpox can be confused with other febrile, rash-causing illnesses, including varicella. Ongoing MPXV and VZV cocirculation likely explains the increasing number of MPXV PCR–negative results, which in turn could affect case definition criteria for sampling clinically suspected cases in the field. Metagenomic NGS identified MPXV–VZV co-infection in the study’s sole fatal mpox case; however, whether the co-infection contributed to the patient’s death remains unclear.

Other limitations of our study include presumed underreporting of mpox cases and suboptimal genotyping because of limited sequencing resources. Local, intracountry, and joint regional efforts for cross-border surveillance are essential for curbing the spread of mpox. Cocirculation of MPXV and VZV highlights the need to adhere to clinical case definitions but also incorporate molecular testing to distinguish similarly manifesting infections.﻿﻿

Appendix 1 Additional information about cocirculation of human monkeypox virus clade 1b with varicella zoster virus, Uganda.

Appendix 2 Additional data from study of cocirculation of human monkeypox virus clade 1b with varicella zoster virus, Uganda.
