# Supplementary material for: Cocirculation of Human Monkeypox Virus Clade 1b with Varicella Zoster Virus, Uganda
Source: Emerg Infect Dis. 2026 Oct;32(10):1756–9. doi: 10.3201/eid3210.251264 (PMC13632837; doi:10.3201/eid3210.251264)
Supplement: Supplementary file 1 [file 25-1264-Techapp-s1.pdf]

Article DOI: https://doi.org/10.3201/eid3210.251264

EID cannot ensure accessibility for supplementary materials supplied by authors. Readers who have difficulty accessing supplementary content should contact the authors for assistance.

Cocirculation of Human Monkeypox Virus Clade 1b with Varicella Zoster Virus, Uganda

Appendix 1

GISAID accessions for MPXV strains contributed from this study

- EPI_ISL_19305614-EPI_ISL_19305615
- EPI_ISL_19587230-EPI_ISL_19587265
- EPI_ISL_19602362-EPI_ISL_19602384
- EPI_ISL_19612226-EPI_ISL_19612237
- EPI_ISL_20047701-EPI_ISL_20047742

Appendix 1 Table 1. Patient demographics. Characteristics of mpox-positive cases and of clinically-suspected individuals testing negative for MPXV by PCR.

Variable	Levels	Mpox positive (N = 115)	Mpox negative (N = 127)
Sex	F	52 (45.2)	59 (46.5)
	M	56 (48.7)	63 (49.6)
	Unknown	7 (6.1)	5 (3.9)
Age groups	0 – 15	8 (7.0)	52 (40.9)
	16 – 30	55 (47.8)	49 (38.6)
	31 – 45	33 (28.7)	12 (9.4)
	>45	5 (4.3)	3 (2.4)
	Unknown	14 (12.1)	11 (8.7)
Region	Central	75 (65.2)	54 (42.5)
	Western	32 (27.8)	34 (26.8)
	Eastern	3 (2.6)	35 (27.6)
	Northern	1 (0.9)	3 (2.4)
	Unknown	4 (3.5)	1 (0.8)
Sampling date	Jul – Sept 24	10 (8.7)	74 (58.3)
	Oct – Dec 24	66 (57.4)	53 (41.7)
	Jan – Feb 25	34 (29.6)	–
	Unknown	5 (4.3)	–
Symptoms	Lymphadenopathy	87 (75.7)	–
	Rash	106 (92.2)	121 (95.3)
	Fever	76 (66.1)	104 (81.9)
	Headache	52 (45.2)	107 (84.3)
	Body weakness	40 (34.8)	84 (66.1)

Appendix 1 Table 2. APOBEC and non-APOBEC mutations in Ugandan MPXV strains.

Mutation class	APOBEC status	Mutations (n)	Sequences (n)	Genes (n)
Unique to Ugandan sequences reported in this study	Non-APOBEC	126	34	47
	APOBEC	96	46	55
Unique to other Ugandan sequences from GISAID	Non-APOBEC	37	9	23
	APOBEC	16	11	14
Shared: present in both study and GISAID sequences	Non-APOBEC	3	3	0
	APOBEC	6	6	1
Total		284		

Using Squirrel software, we inferred a maximum-likelihood phylogeny via IQ-TREE2 and performed ancestral state reconstruction to map APOBEC and non-APOBEC mutation events onto individual branches. This analysis was performed on 137 Ugandan MPXV sequences (115 sequences reported in this study and 22 sequences from GISAID) and on the Nextclade MPXV clade I reference sequence (DQ011155.1). Squirrel outputs a CSV file with branch-specific mutation events, classified as APOBEC or non-APOBEC, and each row in the CSV file represents one mutation observation in one sequence. This was filtered to retain only those occurring on branches leading to Ugandan sequences (284 mutations), of which 118 mutations (41.4%) were APOBEC3-mediated. Mutations were then assigned to one of three classes: unique to Ugandan sequences reported in this study, unique to other Ugandan sequences from GISAID, or shared (present in both groups). Gene-level functional annotations were performed in reference to the UniProtKB. Majority of APOBEC3-mediated mutations were unique to Ugandan sequences generated in this study (n = 96), identified in 46 sequences, spanning 55 genes. In comparison, APOBEC mutations unique to Ugandan sequences from GISAID were fewer (n = 16), identified in 11 sequences across 14 genes. Only a small number of APOBEC mutations were shared between the study and GISAID datasets (n = 6), observed in 6 sequences.
